# Poor sleep health and quality of life among caregivers of patients with prostate cancer

**DOI:** 10.1002/bco2.157

**Published:** 2022-06-03

**Authors:** Sameer Thakker, Rebecca Robbins, Patricia Carter, Girardin Jean‐Louis, Katherine Siu, Tatiana Sanchez Nolasco, Nataliya Byrne, Stephanie L. Orstad, Akya Myrie, Stacy Loeb

**Affiliations:** ^1^ NYU Grossman School of Medicine New York New York USA; ^2^ Division of Sleep Medicine Harvard Medical School Boston Massachusetts USA; ^3^ Department of Medicine, Division of Sleep and Circadian Disorders Brigham & Women's Hospital Boston Massachusetts USA; ^4^ Capstone College of Nursing University of Alabama Tuscaloosa Alabama USA; ^5^ Departments of Psychiatry and Neurology, Miller School of Medicine University of Miami Miami Florida USA; ^6^ Departments of Urology and Population Health NYU Langone Health New York New York USA; ^7^ Department of Medicine, Division of General Internal Medicine and Clinical Innovation NYU Grossman School of Medicine New York New York USA; ^8^ Department of Urology Cleveland Clinic Cleveland Ohio USA

Undergoing cancer treatment can drastically change a patient's quality of life.^1^ Balancing the physical, emotional and financial needs of a patient with cancer can cause significant distress for family members/caregivers, who support the patient through the treatment process.^2^

Previous studies have shown high rates of sleep disturbances and hypnotic medication use among cancer survivors.^3^ For instance, patients with prostate cancer (PCa) may suffer from increased nocturia, other lower urinary tract symptoms, hot flashes, pain, anxiety and depression that present additional challenges to optimal sleep.^4^

Although prior studies have demonstrated that being a caregiver is associated with poor health outcomes across multiple domains, few studies have specifically studied quality of life among caregivers of patients with PCa or the impact of caregiving on sleep.^5^ There are many potential reasons why caregivers to men with PCa may experience sleep disturbances, including psychological and physical stress. Additionally, disturbed sleep among patients with PCa may compound the underlying disruption in sleep for caregivers who are bed partners with the patient with PCa.

The goal of this study was to characterize the prevalence of sleep disturbances and quality of life among a sample of caregivers of patients. We hypothesized that caregivers for patients with PCa likely suffer from poor sleep quality and are at high risk for sleep disorders. These findings will indicate the types of interventions that may benefit this population.

Following IRB approval, an online survey was distributed to caregivers of patients with prostate cancer via multiple prostate cancer foundations and support groups (Us TOO International, the Prostate Cancer Foundation, Fans for the Cure and ZERO Cancer). The survey included demographic questions and multiple validated sleep instruments: the Insomnia Severity Index (ISI), the STOP‐BANG (measuring sleep apnoea risk), the Pittsburgh Sleep Quality Index (PSQI, measuring overall sleep quality) and the Sleep Hygiene Index (SHI, measuring healthy sleep behaviours). The survey also included the validated Caregiver Quality of Life Index – 2Cancer (CQOLC) scale (where lower scores indicate worse quality of life) and the Short Form‐12 survey (SF‐12, measuring mental and physical wellbeing standardized to a score of ‘50’ to represent collated data from the general US population). Respondents were allowed to skip survey questions. In an effort to limit the effects of nonresponse bias, descriptive statistics were calculated independently for each instrument as opposed to an aggregate assessment of the overall cohort. Missing data for each instrument were handled in a two‐step fashion. Mean substitution was first attempted for each case. If imputation was not possible due to sparsity of data for a given response, listwise deletion was applied to exclude responses from the analysis to limit centring bias and ensure that complete‐case analysis could be performed.

Eighty‐one caregivers of patients with PCa completed the survey, the median age was 64 (IQR 56–70). Most caregivers were married to the patient (93%) and slept in the same room as the patient (82%). The median score on the SF‐12 for physical health was 52 (IQR: 40–59) and 38 (IQR: 33–42) on the SF‐12 for mental health (indicating overall worse mental well‐being compared with standardized data from the general population). The median score for the CQOLC scale was 56, suggesting high levels of caregiver stress in the study population.

Figure 1 summarizes the results for sleep among PCa caregivers. On the PSQI, 77% of the respondents met the cutoff for ‘poor sleep’ quality (score >5). On the ISI, 26% met the cutoff for clinical insomnia (score >14). On the STOP‐BANG assessment, 25% were at an elevated risk for sleep apnoea. Finally, the median score was 12 (IQR: 9–16) on the SHI, and 22% scored >17 suggesting poor sleep behaviour. Furthermore, 43% reported using medications (either over the counter or prescribed) at least once a week to help them fall asleep.

**FIGURE 1:**
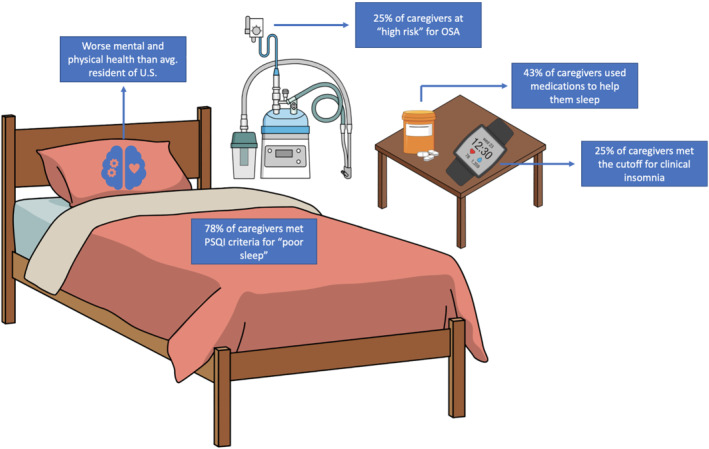
Sleep health infographic summarizing key statistics. Note: Caregivers were offered the option to skip questions, resulting in missing items for the following surveys: SF‐12 (*n* = 12), Caregiver Burden Scale (*n* = 40), Sleep Hygiene Index (*n* = 40), Insomnia Severity Index (*n* = 28), Pittsburgh Sleep Quality Index (*n* = 29) and STOP‐BANG (*n* = 32). Missing responses were extrapolated with regression to the mean or case‐wise exclusion if that was not possible

Our results support the hypothesis that poor sleep quality is prevalent in caregivers of patients with PCa, and they may be at increased risk for undiagnosed sleep disorders such as insomnia or sleep apnoea. Additionally, we found substantial use of sleep aids among caregivers, highlighting the potential for future research to develop methods for improving awareness about sleep disorders and healthy sleep practices.

Prior studies have shown that caregivers experience high rates of chronic illness and report increased levels of medication use and hospitalization likely due to de‐prioritization of their own health during the caregiving process.^6^ These combined findings suggest the importance of addressing undiagnosed sleep disorders and sleep difficulties among caregivers of patients with PCa.

Additionally, we found that caregivers of patients with PCa had worse psychological well‐being than the general population and experienced high levels of caregiver stress. This corroborates previous research showing higher rates of depression and anxiety among PCa caregivers, stemming from feelings of guilt, concerns about treatment outcomes, and financial worries.^7^ Further research is warranted to examine whether improvements in the management of sleep disorders and healthy sleep practices can enhance quality of life among cancer caregivers.

This study is unique in that it is one of the first analyses to examine quality of life among caregivers of patients with PCa through the lens of sleep using multiple validated sleep instruments. Additionally, the utilization of caregiver indices that have been validated in PCa, such as the Caregiver Quality of Life Index – Cancer scale, allows for a more tailored assessment as compared with general caregiver scales or those focused on other conditions.

Larger studies are needed to examine different types of caregivers to fully understand the breadth and complexity of caregiver burden in PCa as it relates to sleep health. A limitation of the study is that the survey was distributed through social media networks making it difficult to assess the true denominator of caregivers who were exposed to the survey link and this online survey can only represent caregivers with internet access, and there may have been selection bias. Even so, our findings of such high rates of sleep problems among caregiver respondents raise a red flag for further work in this area in the clinical and research setting. Additionally, a certain degree of nonresponse bias is expected as participants were allowed to skip questions requiring imputation during analysis. Furthermore, the lack of detailed caregiver characteristic data limits the interpretability of these findings in terms of qualitative comparisons with the general public or other cancer caregivers. Although comparative analysis was not within the scope of this paper, further analysis is needed in order to fully characterize the online caregiver community to better contextualize QoL and outcomes data. Despite these limitations, our results provide novel data, which demonstrate a clear need for increased caregiver support with a specific focus on sleep health.

In conclusion, we found high rates of insomnia, high risk of sleep apnoea, poor sleep quality and poor quality of life among caregivers of patients with PCa. Further research is needed to identify the drivers of poor sleep health and quality of life in caregivers of patients with PCa and subsequently into interventions to improve sleep health and well‐being for caregivers.

## CONFLICT OF INTERESTS

The authors report no relevant financial conflict of interests.
